# Impact of Immersion Media on Physical Properties and Bioactivity of Epoxy Resin-Based and Bioceramic Endodontic Sealers

**DOI:** 10.3390/polym14040729

**Published:** 2022-02-14

**Authors:** Thais Gomes de Moraes, Alan Silva de Menezes, Renata Grazziotin-Soares, Rafael Ubaldo Moreira e Moraes, Paulo Vitor Campos Ferreira, Ceci Nunes Carvalho, Jose Bauer, Edilausson Moreno Carvalho

**Affiliations:** 1 Dentistry Biomaterials Laboratory (Biomma), School of Dentistry, Federal University of Maranhão (UFMA), São Luís 65080-805, Brazil; thais_gomes3@hotmail.com; 2 Department of Physics, Federal University of Maranhão (UFMA), São Luís 65075-120, Brazil; alan.menezes@ufma.br; 3 College of Dentistry, University of Saskatchewan, Saskatoon, SK S7N 5E4, Canada; regrazziotin@gmail.com; 4 Department of Restorative Dentistry, School of Dentistry, University Ceuma (UNICEUMA), São Luís 65065-470, Brazil; rafael.ubaldo@outlook.com (R.U.M.e.M.); ceci.carvalho@ceuma.br (C.N.C.); edilausson.carvalho@ceuma.br (E.M.C.); 5 Dental Materials Division, Department of Restorative Dentistry, School of Dentistry, University of Campinas (UNICAMP), Piracicaba 13414-903, Brazil; paulocampos.vf@gmail.com

**Keywords:** bioceramic sealers, immersion media, physicochemical properties

## Abstract

This study assessed the effects of immersion media [distilled water (dw), phosphate buffered saline (pbs) and simulated body fluid (sbf)] in the physical properties [fluid uptake/sorption/solubility and alkalinization activity (pH)] and bioactivity of a bioceramic sealer: the BioRoot RCS (BioRoot) (Septodont). The epoxy-resin sealer AH Plus (Dentsply) was used as comparison. Sealers were immersed in dw, pbs and sbf to evaluate the fluid uptake/sorption/solubility and pH’s media. Bioactivity was assessed with SEM/EDS, FTIR-ATR and XRD. BioRoot solubility was as follows: sbf > pbs = dw. BioRoot had alkaline pH, and AH Plus had neutral pH, regardless of the medium. BioRoot presented mineral precipitates and peaks indicating hydroxyapatite-precursors in pbs and sbf. AH Plus physical properties were not affected by immersion media and it had no bioactivity. pbs and sbf should be preferred to investigate bioceramic sealers over distilled water, because they were able to highlight the sealer properties. BioRoot maintained the alkaline environment and favored hard tissue deposition.

## 1. Introduction

In Dentistry, an endodontic sealer is one of the components of the filling material—which is used to obturate root canals of teeth that were submitted to root canal treatment. The filling material, when placed inside the tooth’s root canal, acts as a physical barrier against the coronal and periapical leakage [1,2,3] and, it also acts as an alkalinizing substance, which provides its antimicrobial properties [4]. Bioceramic sealers are calcium phosphate-based materials containing bioactive glasses [5,6,7]. The advantage of bioceramic sealers over conventional endodontic sealers, AH Plus for instance, is their improved biological properties, mainly, the bioactivity potential [8,9].

The literature has showed that bioceramic sealers also present a negative feature, the high solubility when immersed in aqueous media [10]. This is an unwelcome feature, because the degradation/dissolution of a sealer may compromise the quality of the seal in the root canal treated tooth, leading to microleakage [11,12]. Nevertheless, some researchers have claimed that the degradation of a bioceramic sealer would help the releasing of compounds that may act as modulators for the periapical tissues. Upon sealer extrusion to the periapical tissues during the obturation procedures (direct contact), or even upon sealer diffusion (indirect influence), the presence of bioactive glasses in the material composition may stimulate the healing/mineralization in the presence of tissue fluids [9].

The standard solubility degree for endodontic sealers is a maximum of 3%-mass loss when immersed in distilled water (ISO 6876:2012) [13]. However, in dentistry, the challenge for researchers is that distilled water does not represent the clinical environment in which an endodontic sealer is exposed to. Because of this, alternative immersion media for testing sealers properties have been used, such as, the phosphate-buffered saline. Bioceramic sealers are less soluble and presented more hydroxyapatite precipitation in their surface when immersed in phosphate-buffered saline, in comparison with the distilled water-immersion [14,15,16]. The lower solubility of bioceramic sealers, when incubated in phosphate-buffered saline, is attributed to the hydroxyapatite precipitates—that would be responsible for filling the voids created by the solubility [15].

This current study tested if simulated body fluid (a medium that reproduces the clinical environment) [17] could emphasize the adequate properties of bioceramic sealers without overestimate the solubility. Therefore, the aim of this study was to assess the effects of different immersion media (90-days immersion in distilled water, phosphate-buffered saline or simulated body fluid) in the physical properties tests [fluid uptake/sorption/solubility and alkalinization activity (pH)] and bioactivity of a bioceramic sealer [BioRoot RCS (BioRoot, Septodont, St Maur-des-Fosses, France)], using AH as comparison [AH Plus (Dentsply De Trey, Konstanz, Germany)]. The null hypothesis was that physical properties and bioactivity potential of BioRoot would not be affected regardless the immersion media—the same would occur for AH Plus.

## 2. Materials and Methods

### 2.1. Sealers and Immersion Media

This study used two endodontic sealers: a bioceramic-calcium silicate based, the BioRoot RCS, and an epoxy resin-based sealer, the AH Plus. The descriptions and characteristics of each sealer are shown in Table 1.

The study used different storage media: (1) distilled water (dw) as the immersion medium (standard medium), (2) phosphate buffered saline (pbs), commercial kit (Sigma Aldrich, São Paulo, Brazil), composed of sodium chloride (NaCl), sodium phosphate (NaHPO), sodium phosphate monobasic (NaH_2_PO_4_ + H_2_O) and magnesium chloride (MgCl_2_); and (3) simulated body fluid (sbf), which was manipulated according to the methodology described by Kokubo & Takadama [17].

For the production of sbf the following reagents were used: sodium chloride (NaCl), sodium hydrogen carbonate (NaHCO_3_), potassium chloride (KCl), dipotassium hydrogen phosphate trihydrate (K_2_HPO_4_·3H_2_O), magnesium chloride hexahydrate (MgCl_2_·6H_2_O), calcium chloride (CaCl_2_), sodium sulfate (Na_2_SO_4_), and tris-hydroxymethyl aminomethane (TRIS, ((HOCH_2_)_3_CNH_2_). These components were dissolved at controlled temperature (36.5 ± 0.5 °C) and pH (7.4 ± 0.05) to avoid spontaneous precipitation.

Finally, the pH was adjusted to 7.4 using a 1 M hydrochloric acid (HCl) solution. Throughout the process, it was confirmed that the solution remained colorless and left no deposits in the container. SBF was inserted in a plastic container and stored in a refrigerator at 4 °C.

### 2.2. Specimen Preparation and Aging

Twenty-four cylindrical specimens of each sealer were prepared and divided into 3 experimental groups for each sealer (*n* = 8), according to the immersion media. A metal matrix (10 mm diameter and 1 mm height) was used to fabricate the samples. Specimens were initially incubated for 7 days at 37 °C and 95–100% humidity to allow complete setting before testing [18,19,20]. After that, specimens were lightly washed, dried and placed in a desiccant dehumidifier for 30 days. Then, they were weighed three times and the average weight (W_1_) recorded. New weight measurements were made at 24 h, 48 h, 7 days, 30 days, 60 days and 90 days (W_2_). After each weight measurement the immersion medium was replaced. After the 90 days, the specimens were stored in the desiccant dehumidifier for more 30 days and then, weighted to get the final weight measurement (W_3_).

### 2.3. Fluid Uptake, Sorption, Solubility and Alkalinization Activity (pH)

The difference between the initial weight and the final weight (W_1_–W_3_) were recorded to the value close to 0.0001 g. The difference in weight was calculated as a percentage (%) of the original weight of the material, recorded to the value close to 0.001% [15,20,21]. The volume (V) was established as a constant for all the specimens and was calculated with the formula:

V = π × r^2^ × h, where r = radio and h = height

Fluid uptake was defined as the specimen weight variation occurred between W_2_ (24 h, 48 h, 7 d, 30 d, 60 d and 90 d) in relation to the specimen initial weight (W_1_).

Fluid uptake was calculated with the formula:

$$W_{2}=W_{1}/V$$

Sorption and solubility (μg × mm^3^) values were calculated with the following formulas [22]:

$$\mathrm{Sorption}=W_{2}-W_{3}/V$$

$$\mathrm{Solubility}=W_{1}-W_{3}/V$$

The initial pH value of each immersion media (dw, pbs, sbf) was measured using a digital pH meter. The following measurements were performed alongside with the fluid uptake/sorption/solubility tests. After each measurement the pH meter was carefully cleaned with deionized water and dried with absorbent paper.

### 2.4. Bioactivity (SEM/EDS, FTIR-ATR and XRD Analyses)

Two samples from each experimental group were analyzed at SEM/EDS (TM3030, Hitachi, Japan) to verify the presence of mineral precipitates. Specimens of BioRoot and AH Plus not immersed in any solution were used as controls. A Fourier Transform Infrared Spectrophotometer/Attenuated Total Reflection (FTIR-ATR) (IRTracer-100, Shimadzu, Kyoto, Japan) characterized the bands for calcium and phosphate. The spectra were obtained using in transmittance-method, 40 scans with 4 cm^−1^ resolution and 400–4000 cm^−1^ range—with tables of potassium bromide. Additional characterization of mineral precipitates was performed with X-ray Diffraction (XRD) and Scattering analysis (D8 Advance, Bruker, Karlsruhe, Germany), using Cu-Kα radiation (40 kV, 40 mA) with a linear detector/0.6 mm slit, at 25 °C, in a 2θ range from 9 to 79° with a step size of 0.02°.

### 2.5. Data Analysis

The values from fluid uptake/sorption/solubility tests were compared (immersion medium vs. sealer) using Two-way ANOVA and Holm-Sidak (α = 0.05), for contrast of means (SigmaPlot v. 13.0, Systat Software Inc., San Jose, CA, USA). pH and bioactivity results were descriptively reported.

## 3. Results

BioRoot presented the following results: (i) higher fluid uptake when immersed in pbs (*p* < 0.05), when compared to the dw and sbf (*p* > 0.05) where it had negative values at 90 days; (ii) higher sorption when immersed in pbs (*p* < 0.05) compared to the dw and sbf (*p* > 0.05); and (iii) higher solubility when immersed in sbf (*p* < 0.001) compared to the dw and pbs (*p* = 0.053). The immersion medium did not affect the uptake of fluid, sorption or solubility for AH Plus (*p* > 0.05) (Table 2, Table 3 and Table 4).

**Figure 1:**
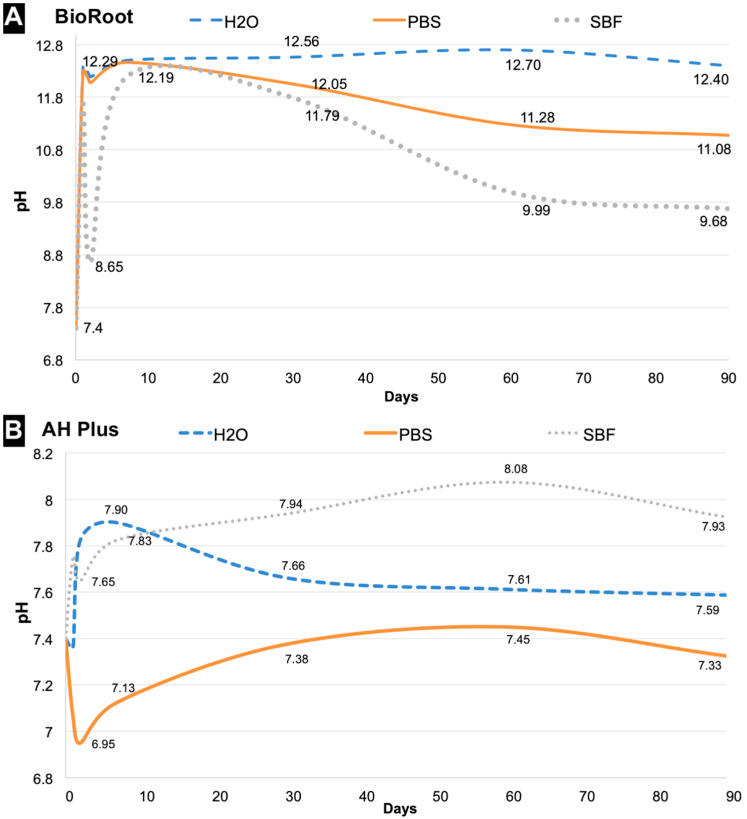
Results of pH for (**A**) BioRoot and (**B**) AH sealers in different immersion media [Distilled water (H_2_O), PBS and SBF] over different time interval.

BioRoot had alkaline pH at all time-intervals, regardless of the immersion medium. When immersed in sbf, the pH dropped at 48 h (pH = ~8.5) (Figure 1A). AH Plus had pH levels close to neutral at all time-intervals, regardless of the immersion medium (Figure 1B). 

**Figure 2:**
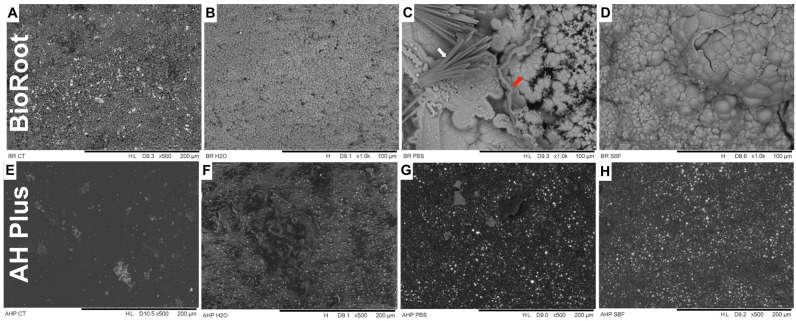
**On the top**: SEM images for BioRoot sealer (×1000, 100 μm scale), (**A**): BioRoot control group, (**B**): BioRoot after 90 days immersed in distilled water, (**C**): BioRoot after 90 days immersed in pbs, (**D**): BioRoot after 90 days immersed in sbf. White arrow: needle-like precipitates, red arrow: spherical precipitates. **On the bottom**: SEM images for AH sealer (×500, 100 μm scale), (**E**): AH control group, (**F**): AH after 90 days immersed in distilled water, (**G**): AH after 90 days immersed in pbs, (**H**): AH after 90 days immersed in sbf.

BioRoot, under SEM analysis, presented many needle-like and spherical precipitates on the surface after being immersed in pbs (Figure 2C), where multiple shapes can be noticed on the material surface compared to the neat material (A) and to the material immersed in water (B). BioRoot had some spherical precipitates after being immersed in sbf (Figure 2D); and absence of precipitates after being immersed in dw (Figure 2B). EDS analysis found high percentages of elements Calcium (Ca) and Phosphor (P) for the samples that were immersed in PBS and SBF. SEM images for AH Plus sealer showed absence of mineral precipitates regardless of the immersion medium (Figure 2E–H). No different shapes were noticed and the material surface remained relatively unchanged. EDS analysis found only elements that are part of the material composition.

**Figure 3:**
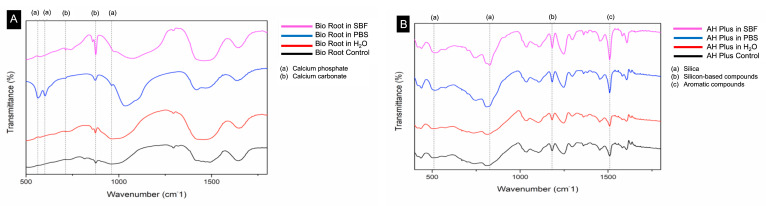
FTIR spectra for BioRoot (**A**) and AH Plus (**B**) after 90-days immersed in distilled water, pbs, sbf and control group. The following peak values were found for BioRoot: (a) calcium phosphate 560 cm^−1^, 600 cm^−1^, and 960 cm^−1^, and (b) calcium carbonate 714 cm^−1^, and 874 cm^−1^. The following peak values were found for AH: (a) silica 509 cm^−1^, and 822 cm^−1^, (b) silicon-based compound 1.181 cm^−1^, and (c) aromatic compounds, 1.512 cm^−1^.

BioRoot, under FTIR-ATR analysis, found peaks indicating components of hydroxyapatite-precursors (Figure 3). After being immersed in pbf and sbf, BioRoot had more significant peaks corresponding to the calcium phosphate and calcium carbonate (Figure 3). (a) calcium phosphate 560 cm^−1^, 600 cm^−1^, and 960 cm^−1^, and (b) calcium carbonate 714 cm^−1^, and 874 cm^−1^. After being immersed in dw, BioRoot had only one peak corresponding to the calcium carbonate (874 cm^−1^). In contrast, for AH Plus sealer the FTIR-ATR analysis found absence of peaks precursors of bioactivity. Peaks were attributed to the silica, a silicon-based compound and aromatic compounds: (a) silica 509 cm^−1^, and 822 cm^−1^, (b) silicon-based compound 1.181 cm^−1^, and (c) aromatic compounds, 1.512 cm^−1^ (Figure 3). 

**Figure 4:**
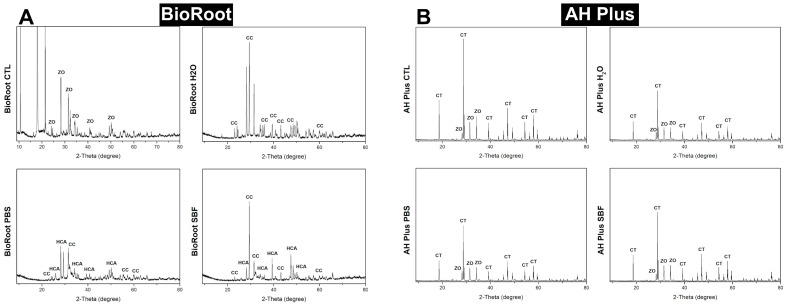
XRD spectra for BioRoot (**A**) and AH Plus (**B**) after 90-days immersed in distilled water, pbs, sbf and control group. For BioRoot: calcium carbonate (CC), carbonated hydroxyapatite (HCA) peaks were identified in the samples that were immersed in pbs and sbf. For AH: zirconium oxide (ZO) and calcium tungstate (CT) peaks were identified for all groups.

BioRoot, under XRD spectra, presented significant presence of calcium carbonate (CC) and carbonated-hydroxyapatite (HCA) after samples being immersed in pbs and sbf. After being immersed in dw, calcium carbonate (CC) peaks were identified, but no carbonated-hydroxyapatite (HCA) peaks. XRD spectra for AH Plus sealer presented zirconium oxide (ZO) and calcium tungstate (CT) peaks, regardless of the immersion media; those of which are part of the material composition (Figure 4). Also, it had absence of hydroxyapatite-precursors peaks (absence of CC and HCA in the AH Plus spectra) (Figure 4).

## 4. Discussion

This study investigated sbf as an alternative immersion medium for testing physical properties and bioactivity of a bioceramic sealer. Sbf was assumed to be a good alternative medium (in comparison to dw and pbs) because it not only would recreate the clinical environment [17] but also it could emphasize the adequate properties of BioRoot: its bioactivity potential and less solubility.

However, contrary to our expectations, sbf made BioRoot much more soluble than the other immersion media. As desired, BioRoot had alkaline capacity in any immersion media and presented bioactivity when immersed in pbs and sbf. AH Plus physical properties were not affected in different immersion media and it did not present any bioactivity. The results of this study rejected the null hypothesis because physical properties and bioactivity potential of BioRoot were affected depending on the immersion medium—differently than what occurred with AH Plus.

AH had lower values of fluid uptake/sorption/solubility [11,14,15,20,23,24]. BioRoot had 19.87% of mass loss (solubility) at 90 d immersed in dw. A slight reduction in mass loss (16.83%) was observed when BioRoot was immersed in pbs, but without statistical difference. When the bioceramic sealer was immersed in sbf, it had the highest mass loss: 32.39%. Another study also found 15.8% of mass loss (28 d) when BioRoot was incubated in dw and 30.2% when incubated in HBSS (Hank’s balanced salt solution) that is also a medium used to reproduce body fluids [16].

It is known that when a solid is added to the sbf immersion medium the composition of the solution may be altered—consequently altering also the saturation level of the solution- and this might result in a modification in the place where precipitates are formed. In other words, the formation of precipitates may occur in the solid surface or into solution [25]. Another cause for the modification in the location of precipitates is, naturally, the immersion of a solid capable of releasing a high amount of calcium and phosphate ions into the medium, and this was what occurred with BioRoot into the sbf. To better understand this process, the literature has explained that the 2-amino-2 (hydroxymethyl)-1,3-propanediol, a compound named ‘TRIS’ that is present in the sbf medium, is able to get attached to the calcium ions (derived from the sealer). These calcium ions, therefore, become unavailable to precipitate as calcium phosphate. The same process also occurs with other sbf-compounds such as amines and hydroxyl groups. The higher the concentration of these compounds, the lower the ability of the solid to precipitate/form hydroxyapatite [26]. Supported by the previous statements, an explanation for the observed high mass loss of BioRoot in sbf, is that during the ionic exchange between sealer-medium, there was an imbalance imposed to the environment, leading to the sealer dissolution—justifying, then, the high solubility/mass loss. The precipitation might have occurred not only in the sealer surface (as showed in the SEM images), but also into the solution.

Two differential aspects of this study were (i) the long-time immersion of the specimens (90 d in media); and (ii) the placement of the specimens in the desiccant dehumidifier before and after the immersion. The majority of the solubility studies have incubated the specimens for only 30 d [8,10,11,16,17,20]. We know that the desiccation has a compelling effect on fluid uptake/sorption/solubility of endodontic bioceramic sealers [20,22]. Previous studies have placed the specimens in the desiccant only after the immersion period—which could overestimate the sealer solubility. This occurs because the water that is not incorporated into the specimen during the immersion period (hydration of the solid) evaporates into the desiccator, increasing the values of mass loss [27].

When BioRoot was incubated for 90 d in dw and sbf, it had negative values of fluid uptake, meaning that the dissolution of the sealer in the medium was higher than its capacity of absorb fluids. Differently, when BioRoot was incubated in pbs, the weight of the specimen after immersion-periods was, on average, +17.40% higher than the initial mass. Other studies that used micro-computed tomography have explained that high solubility values do not necessarily mean that a morphologic alteration will occur in the specimen [22,28]. One study investigated two bioceramic sealers (TotalFill BC Sealer e Bio-C Sealer) and found less than 2% of volumetric alteration and more than 10% of solubility [22]. This happened because those sealers presented expansion and mass loss concomitantly—i.e., the solubility was compensated by the fluid uptake [28].

The alkaline capacity (pH) is related to the dissolution and ionic releasing of the sealers. This study showed that the high solubility of BioRoot fostered alkaline pH values. When immersed in sbf, the initial pH was 7.4, increasing to 11.67 (at day 1) and, further dropping to 8.65 (at day 2). After this variation, the pH increased again and then, stabilized (Figure 1). This reduction in pH of BioRoot around the 2nd day after immersion in sbf needs further investigation. One plausible explanation for this would be the formation of transitorily chemical compounds with acidic profile (formed approximately 48 h after sealer immersion). This acid-like compounds could have dropped the pH—and they were dissolved after day 2. We hypothesized this because the observed turbidity in the solution around the day 2, which disappeared soon after.

In contrast, the AH Plus negative values of solubility (in all immersion media) fostered the neutral pH. Overall, pH values found in this study were in agreement with values reported in the current literature [10,12,14,15]. In a clinic situation, after the root canal obturation, if the BioRoot is able to long-term maintain the environment alkaline, the healing in the periapical area will be favored and this is particularly important in cases where the pH was reduced due to an inflammatory process. Also, the BioRoot alkaline pH will have antimicrobial effects and will favor the hard tissue deposition [29,30,31,32].

BioRoot presented bioactivity potential after 90 d incubated in pbs and sbf, but not in dw. SEM analysis for BioRoot expressed evident formation of very defined and diverse precipitates (needle-like + spherical) when incubated pbs; and a little less defined and uniform (spherical) when incubated in sbf. The elemental analysis (SEM/EDS) confirmed the higher concentration of calcium and phosphate in the precipitates, according to the literature [8,27,33,34,35]. Under FTIR/ATR and XRD analyses, BioRoot presented expressive peaks of calcium carbonate and carbonated hydroxyapatite when incubated in pbs and sbf; endorsing the findings from SEM analysis. However, when BioRoot was incubated in dw, even with the absence of precipitates on the material surface (SEM analysis), peaks of calcium carbonate were found in the FTIR/ATR and XRD spectra (Figure 3 and Figure 4). The different findings between the analyses are due to the sensibility of the used tools: FTIR/ATR and XRD are more sensitive to identify hydroxyapatite-precursors [5]. Previous authors [36] also showed that FTIR vibrational features of the modified sealers preserved all the fingerprints of the blended components. In addition, the main peaks of the hydroxyapatite precursors groups are visible at the same wavenumbers as in the spectrum of neat sealer and the intensity of the bands at 572 cm^−1^, 1020 and 1237 cm^−1^ are enhanced. Then, as calcium carbonated can precedes hydroxyapatite formation; this fact could be an indication of a great potential of BioRoot to induce/conduct hard tissue formation (even in dw and this hypothesis deserve to be further investigated.

The main limitation of this study is the impossibility to directly transport the findings to the clinical setting, as any other laboratorial experiment [37]. However, our results can guide the decision-making process for choosing a sealer; in addition to possibly inspire further in vivo investigations. Our findings led to the understanding that, the tested bioceramic sealer is a superlative option to obturate root canals of patients that presented necrotic teeth with apical periodontitis. The BioRoot high pH and bioactivity potential are assets in those cases, since these properties will make the area improper to infection and will stimulate healing. BioRoot solubility (sbf > pbs = dw) might be somehow convenient, since its alkaline capacity and bioactivity are linked to the ionic releasing, which is prompted by the solubility.

## 5. Conclusions

The importance of our results and the main outcome of this study from the practical perspective is that pbs and sbf should be preferred to research bioceramic sealers’ properties over distilled water. It was showed that the most appropriate immersion medium to test physical properties and bioactivity of endodontic bioceramic sealers (BioRoot) was the pbs—a medium that was able to highlight the sealer properties: high pH, strong evidence of bioactivity potential, and solubility a little lower than that produced by immersion in sbf. Therefore, the practical relevance of these findings is that scientists may use pbs and sbf to guarantee the production of reliable and accurate results when investigating physical properties of commercial bioceramic products. Bioroot showed formation of hydroxyapatite nanoprecursor compounds only in sbf and pbs media. The AH Plus did not show the formation of hydroxyapatite precursors, but it showed excellent chemical stability independent of the storage medium.

## Figures and Tables

**Table 1:** Manufacturer, batch number and handling instruction for the endodontic sealer used in this study: BioRoot RCS and AH Plus.

Material (Manufacturer) (Batch Number)	Composition	Presentation and Handling Instructions
BioRoot RCS (Septodont, St Maur-des-Fosses, France) (B23970)	Tricalcium silicate, zirconium oxide, water, calcium chloride, water-soluble polymer.	Powder/liquid Proportion: 1 spoon of powder to 5 drops of liquid, mixed in a glass plate, for 1 min.
AH Plus (Dentsply De Trey, Konstanz, Germany) (364801L)	Paste A: bisphenol A ether, diglycidyl, calcium tungstenate, zirconium oxide, aerosil and iron oxide. Paste B: adamantan amine, *n*-dibenzyl-5-oxanonane-diamine, calcium tungstenate, zirconium oxide and silicone oil.	Base paste and catalyst paste dispersed on a glass plate in a ratio of 1:1, to obtain a homogeneous paste.

**Table 2:** Results of fluid uptake analysis in absolute values (µg/mm^3^) and percentage (%) for BioRoot and AH Plus sealers when immersed in dw, pbs and sbf *.

	Sorption			
	AH Plus		BioRoot	
	(µg/mm^3^)	(%)	(µg/mm^3^)	(%)
**dw**	39.01 (20.70) Ab	1.48	462.58 (39.86) Ca	17.55
**pbs**	29.46 (13.01) Ab	1.13	872.29 (81.88) Aa	34.23
**sbf**	22.29 (6.50) Ab	0.81	668.15 (24.59) Ba	26.61

* Different capital letters in the same column represent significant differences for the same sealer, *p* < 0.05. Different lower letters in the same line represent significant differences between sealers, *p* < 0.05.

**Table 3:** Results of sorption analysis in absolute values (µg/mm^3^) and percentage (%) for BioRoot and AH sealers when immersed in dw, pbs and sbf *.

	Solubility			
	AH Plus		BioRoot	
	(µg/mm^3^)	(%)	(µg/mm^3^)	(%)
**dw**	−3.66 (4.29) Ab	−0.15	523.73 (39.86) Ba	19.87
**pbs**	−6.85 (3.97) Ab	−0.24	428.18 (81.88) Ba	16.83
**sbf**	−6.84 (2.62) Ab	−0.36	818.63 (24.59) Aa	32.39

* Different capital letters in the same column represent significant differences for the same sealer, *p* < 0.05. Different lower letters in the same line represent significant differences between sealers, *p* < 0.05.

**Table 4:** Results of solubility analysis in absolute values (µg/mm^3^) and percentage (%) for BioRoot and AH sealers when immersed in dw, pbs and sbf *.

	Fluid Uptake			
	AH Plus		BioRoot	
	(µg/mm^3^)	(%)	(µg/mm^3^)	(%)
**dw**	42.68 (19.18) Aa	1.62	−61.15 (14.33) Bb	−2.32
**pbs**	36.31 (12.02) Ab	1.36	444.11 (91.82) Aa	17.40
**sbf**	24.03 (4.90) Aa	0.96	−193.97 (104.69) Cb	−7.54

* Different capital letters in the same column represent significant differences for the same sealer, *p* < 0.05. Different lower letters in the same line represent significant differences between sealers, *p* < 0.05.

## Data Availability

Data is contained within the article.
